# Role of adipose tissue in methionine–choline-deficient model of non-alcoholic steatohepatitis (NASH)^☆^

**DOI:** 10.1016/j.bbadis.2014.02.012

**Published:** 2014-07

**Authors:** Pooja Jha, Astrid Knopf, Harald Koefeler, Michaela Mueller, Carolin Lackner, Gerald Hoefler, Thierry Claudel, Michael Trauner

**Affiliations:** a Hans Popper Laboratory of Molecular Hepatology, Division of Gastroenterology and Hepatology, Department of Internal Medicine III, Medical University of Vienna, Vienna, Austria; b Laboratory of Experimental and Molecular Hepatology, Division of Gastroenterology and Hepatology, Department of Internal Medicine, Medical University of Graz, Graz, Austria; c Institute of Pathology, Medical University of Graz, Graz, Austria; d Core Facility for Mass Spectrometry, Medical University of Graz, Austria

**Keywords:** ATGL, adipose triglyceride lipase, ACC1, acetyl-CoA carboxylase, ACSL1, long-chain fatty-acid-coenzyme A ligase, ap2/FABP4, adipocyte protein 2/fatty acid binding protein 4, ATP5B, ATP synthase, H + transporting mitochondrial F1 complex, beta subunit, BAT, brown adipose tissue, C/EBPα, CCAAT/enhancer-binding protein alpha, CIDEA, cell death-inducing DFFA-like effector A, Cox4i1, cytochrome c oxidase subunit IV isoform 1, CPT1β, carnitine palmitoyltransferase 1β, DNL, de novo lipogenesis, DGAT, diglyceride acyltransferase, FA, fatty acid, FGF21, fibroblast growth factor 21, HSL, hormone sensitive lipase, IHC, immunohistochemistry, MCD, methionine–choline-deficient, NAFLD, non-alcoholic fatty liver disease, NASH, non-alcoholic steatohepatitis, NEFA, non-esterified fatty acid, SD, standard deviation, PCYT1A, choline-phosphate cytidylyltransferase A, PCYT2, ethanolamine-phosphate cytidylyltransferase 2, PEMT, phosphatidylethanolamine N-methyltransferase, PPARγ2, peroxisome proliferator-activated receptor γ2, SCD1, stearoyl-CoA desaturase-1, SNS, sympathetic nervous system, SREBP1c, sterol regulatory element-binding protein 1c, TG, triglyceride, TFA, total fatty acid, UCP-1, uncoupling protein 1, WAT, white adipose tissue. Common name of fatty acids: 14:0 Myristic acid, 16:0, Palmitic acid, 18:0, Stearic acid, 20:0, Aracidic acid, 24:0, Lignoceric acid, 16:1n7, Palmitoleic acid, 18:1n9, Oleic acid, 18:1n11, Vaccenic acid, 18:2n6, Linoleic acid, 20:4n6, Arachidonic acid, 22:4n6, Adrenic acid, 18:3n6, γLinolenic acid, 18:3n3, αLinolenic acid, 20:3n6, Dihomo γlinolenic acid, 20:5n3, Eicosapentaenoic acid (EPA), 22:5n3, Docosapentaenoic acid (DPA), 22:6n3, Docosahexaenoic acid, NAFLD, Lipolysis, Liver, ATGL, HSL, FGF21

## Abstract

Methionine–choline-deficient (MCD) diet is a widely used dietary model of non-alcoholic steatohepatitis (NASH) in rodents. However, the contribution of adipose tissue to MCD-induced steatosis, and inflammation as features of NASH are not fully understood. The goal of this study was to elucidate the role of adipose tissue fatty acid (FA) metabolism, adipogenesis, lipolysis, inflammation and subsequent changes in FA profiles in serum and liver in the pathogenesis of steatohepatitis. We therefore fed ob/ob mice with control or MCD diet for 5 weeks. MCD-feeding increased adipose triglyceride lipase and hormone sensitive lipase activities in all adipose depots which may be attributed to increased systemic FGF21 levels. The highest lipase enzyme activity was exhibited by visceral WAT. Non-esterified fatty acid (NEFA)-18:2n6 was the predominantly elevated FA species in serum and liver of MCD-fed ob/ob mice, while overall serum total fatty acid (TFA) composition was reduced. In contrast, an overall increase of all FA species from TFA pool was found in liver, reflecting the combined effects of increased FA flux to liver, decreased FA oxidation and decrease in lipase activity in liver. NAFLD activity score was increased in liver, while WAT showed no changes and BAT showed even reduced inflammation. Conclusion: This study demonstrates a key role for adipose tissue lipases in the pathogenesis of NASH and provides a comprehensive lipidomic profiling of NEFA and TFA homeostasis in serum and liver. Our findings provide novel mechanistic insights for the role of WAT in progression of MCD-induced liver injury.

## Highlights

• MCD model of NASH increases lipase activity in WAT as a critical determinant of hepatic FA flux and lipotoxicity.
• The maximal increase of ATGL and HSL activity is in visceral WAT.
• Increased lipase activity may be due to enhanced FGF21 signaling.
• NEFA-18:2n6 is preferentially increased in serum and liver due to lipolysis in WAT.
• MCD diet enhances the activity of BAT and diminishes its inflammatory markers.

## 1 Introduction

Non-alcoholic fatty liver disease (NAFLD) includes a disease spectrum ranging from simple fatty liver (steatosis), to non-alcoholic steatohepatitis (NASH), cirrhosis and ultimately hepatocellular carcinoma (HCC) [1]. NAFLD is commonly observed in obese individuals, diabetics and patients with metabolic syndrome [2].

Methionine–choline-deficient (MCD) diet is a commonly used rodent model of NASH which results in hepatic steatosis, oxidative stress, inflammation and fibrosis [3–6]. Liver injury may be significantly attributed to the lack of methionine and choline in the diet. Since choline is essential for de novo synthesis of phosphatidylcholine, required for the export of triglycerides (TG) via very low density lipoprotein (VLDL) packaging, its deficiency causes steatosis [7,8]. Methionine is an essential amino acid that plays a key role in protein synthesis and is an intermediate in synthesis of S-adenosylmethionine (SAM) and glutathione (GSH), two important antioxidants [9]. Therefore methionine deficiency predisposes to oxidative stress, inflammation and fibrosis [8]. Additionally, gut-derived bacterial products play a causal role in the pathogenesis of NASH [5]. MCD diet increases plasma endotoxin levels which enhance steatohepatitis via toll-like receptor-4 signaling and inflammation is diminished in endotoxin resistant MCD-fed mice [6]. Most studies on MCD-induced NASH model have focused largely on the factors pertaining to liver while the role of adipose tissue in the pathogenesis of NASH has not been well understood. A multiple parallel hit model has been proposed for the pathogenesis of NASH where the initial step (first-hit) involves fat accumulation in liver and many hits, especially gut-derived and adipose tissue-derived factors, may act in parallel, resulting in hepatic inflammation [10,11].

There are 3 sources of FAs for hepatic TG accumulation: (i) diet, (ii) de novo lipogenesis (DNL) and (iii) adipose tissue [12–14]. Altered lipid composition in both plasma and liver has been shown in patients with NAFLD and NASH and hence has been implicated in triggering inflammation and serving as endogenous danger signals [15,16]. Additionally, hepatic lipotoxicity may ensue when hepatic capacity to utilize (oxidation and new membrane generation), store and export fatty acids (FAs) in the form of VLDL is overwhelmed by FA flux from the periphery or hepatic DNL [12,14]. According to this concept, pure fatty liver can progress to NASH when adaptive mechanisms protecting hepatocytes from FA-mediated lipotoxicity are overwhelmed [12,17].

Adipose tissue is the main source of FAs in circulation. Obesity, insulin resistance, and NAFLD are all associated with increased adipose tissue lipolysis [12,13]. Additionally, rapid weight loss (e.g. due to significant calorie restriction and bariatric surgery) induces lipolysis in the AT [18,19]. Thus understanding the factors influencing lipolysis may be of significant importance. Adipocyte triglyceride lipase (ATGL) is the rate limiting enzyme in triglyceride hydrolysis [20–22] and utilizes triacylglycerol (TG) as a substrate. ATGL catalyzes the first step of TG breakdown, whereas hormone-sensitive lipase (HSL) subsequently hydrolyses diacylglycerol (DG) into monoacylglycerol (MG). Finally monoacylglycerol lipase (MGL) hydrolyses monoacylglycerol (MG) to FA and glycerol. These three enzymes constitute the canonical pathway for triglyceride breakdown [20–23].

We previously showed that MCD feeding in non-diabetic, non-obese, wild-type (WT) mice for 2 weeks resulted in WAT loss due to increased lipase activity in WAT depots [3]. In the current study, we expand our previous observation to study the role of adipose tissue in the pathogenesis of NASH in ob/ob mouse model (with key features of obesity and insulin resistance) under MCD challenge. Our findings show that lipase activities were elevated in all adipose depots in line with increased FGF21 signaling. This resulted in increased FA flux from adipose tissue to the liver, thereby causing steatosis, inflammation and change in FA homeostasis in serum and liver. While MCD feeding had no effect on inflammatory markers in WAT, in BAT it was significantly decreased due to increased BAT activity.

## 2 Methods

### 2.1 Animal care and use

ob/ob Mice were obtained from Charles River, Germany. All animals were housed under a 12:12-h light/dark cycle and permitted ad libitum consumption of water and diet. Mice were harvested in non-fasted state between 12 and 4 pm. All experimental protocols were approved by the local Animal Care and Use Committees according to criteria outlined in the *Guide for the Care and Use of Laboratory Animals* prepared by the U.S. National Academy of Sciences (National Institutes of Health publication 86-23, revised 1985).

### 2.2 Diet

MCD and its corresponding control diet was obtained from Safe-diets (Augy, France). The diets were identical in all nutrients except methionine (2.8 g/kg) and choline (1.6/kg), which were present in the control diet only. Both diets provided 64.2% kcal as carbohydrate, 25.8% kcal as protein, and 10% kcal as fat.

### 2.3 Serum analysis

Blood was collected by retro-orbital puncture under anesthesia and centrifuged for 15 min at 4500 rpm. Serum was stored at − 80 °C until analysis. ALT, AST, bile acids and bilirubin were analyzed on a Hitachi 917 analyzer (Boehringer Mannheim, Mannheim, Germany). Serum FFAs were determined by using commercial kit (Wako Chemicals, Neuss, Germany) on an Olympus AU640 analyzer (Olympus Diagnostika, Hamburg, Germany). Serum FGF21 was measured by using ELISA kit (Millipore). Plasma glucose was measured by using Accu-Check glucometer (Roche Diagnostics).

### 2.4 Assay for tissue TG hydrolase activity

TG hydrolase assay was performed as previously described [3,24]. In brief, WAT and liver samples of mice were surgically removed and washed in phosphate-buffered saline (PBS), containing 1 mM EDTA and 5 U/ml heparin. Tissues were stored in liquid nitrogen until use. Homogenization was performed on ice in lysis buffer (0.25 M sucrose, 1 mM EDTA, 1:2000 dilution of protease inhibitor cocktail, pH 7.0) using MagNA Lyser (Roche). Tissue lysates were centrifuged twice at 13,000 rpm, 4 °C. The lipid free infranatant (cytosolic fraction) was collected and used for TG hydrolase assays. The substrate for the measurement of TG hydrolase activity containing triolein and [9,10-^3^H]triolein (Hartmann analytic GMBH, Vienna, Austria) as radioactive tracer was emulsified with phosphatidylcholine and phosphatidylinositol in 100 mM potassium phosphate buffer (pH7) using a sonicator, followed by addition of 5% FA free bovine serum albumin. Final substrate concentration was 167 nmol of triolein/assay (8000 cpm of [9, 10-3H]triolein/nmol). 25 μg WAT, 35 μg BAT and 50 μg of liver cytosolic fractions (0.1 ml) supplemented with or without specific inhibitor for HSL(10 μM) (NNC 0076-0000-0079; a kind gift from Novo Nordisk, Denmark) were incubated with 0.1 ml of the substrate in a water bath at 37 °C for 60 min. The reaction was terminated by adding 3.25 ml of methanol/Chloroform/heptane (10:9:7) and 1 ml of 0.1 M potassium carbonate, 0.1 M boric acid, (pH 10.5). After centrifugation at 2300 rpm for 15 min, radioactivity in 1 ml of the upper phase was determined by liquid scintillation counting in LS 6500 Multi-Purpose Scintillation Counter (Beckman Coulter, Fullerton, CA). ATGL activity was defined as the remaining activity not inhibited by the HSL inhibitor (activity in the presence of the HSL inhibitor). HSL activity was calculated by subtracting the activity obtained in the presence of the HSL inhibitor from total activity.

### 2.5 Western blot analysis of cytosolic ATGL and HSL

For ATGL and HSL Western blots, cytosolic extracts were used as described for TG hydrolase activity. Blots were probed with antibodies specific to ATGL (Cell Signaling, cat. no. 2439S; 1:1000), HSL (Cell signaling, cat. no. 4107S; 1:500) and β-Actin (Sigma-Aldrich, cat. no. A5441) as primary antibodies and a peroxidase-conjugated affinity-purified secondary antibody obtained from Cell signaling (cat. no. 7074; 1:3000) for ATGL, and HSL and from Dako (cat. no. P0260; 1:1000) for β-Actin. For quantification of relative expression levels, blots were scanned by using a densitometer (BioRad) and quantified with ImageJ software (NIH). 20 μg and 30 μg of cytosolic extract were used for ATGL and HSL blots for WAT and liver, respectively.

### 2.6 Adipocyte size quantification

Gonadal and BAT fat pads of ob/ob mice fed either control or MCD diet were fixed in 4% buffered formaldehyde and embedded in paraffin. Hematoxylin- and eosin (H&E)-stained WAT (3 μm thick) and BAT (2 μm thick) sections were used to take random pictures (6–10) from different locations at 40 × magnification. The area of each adipocyte was determined by using the Cell Sens Standard 1.8.1 software by manually outlining each cell. On an average 40 and 60 fat cells from WAT and BAT respectively, were measured from each sample for the control group and 70 and 120 from WAT and BAT respectively for the MCD group.

### 2.7 Immunohistochemistry for UCP-1 and F4/80

For detection of UCP-1 positive cells, 3 μm and 2 μm thick paraffin sections of gonadal WAT and BAT respectively were mounted on charged glass slides. Deparaffinization was done in xylene, rehydrated in ethanol, treated with 1 mM sodium EDTA buffer((pH 8.0) in microwave, followed by 1% hydrogen peroxide. Sections were incubated for 1 h with rabbit polyclonal UCP-1 antibody (Abcam, ab10983; 1:100 dilution in antibody diluent (DAKO)). Sections were incubated with Dako Real real Envision Detection System (K5700) for 30 min. Subsequently, detection was carried out by using AEC Substrate Chromogen ready-to-use (Dako K3464) under microscopic control. Negative controls were performed in the absence of primary antibody. Nuclei were counter-stained in Hemalaun nach Mayer for 30 s. Aquatex as glue was used for fixing the cover slips onto the slide.

For detection of F4/80 positive cells, slides were deparaffinized in xylene, reyhdrated in ethanol and treated with 0.1% protease XXIV, followed by 1% hydrogen peroxide. Sections were incubated overnight at 4 °C with rat-anti-mouse F4/80 antibody (AbD Serotec, MCA497GA; 1:50 dilution in antibody diluent (DAKO)). Subsequently, sections were incubated with polyclonal rabbit-anti-rat secondary antibody (DAKO, E0468, dilution 1:100 in antibody diluent (DAKO)) for 30 min followed by incubation with rabbit on rodent HRP-polymer (catalog no. RMR622; Biocare Medical) for 30 min. Subsequently, detection was carried out as stated above. For quantification of F4/80 positive cells, the slides were scanned at 40 × magnification by using scanscope (Aperio technologies, CA). The positive cells were then manually quantified in 10, 40 × power fields from each sample.

### 2.8 GC–NICI/MS of free fatty acids

Mouse livers (50–70 mg) were homogenized in 0.5 ml of PBS and 0.5 ml of methanol. Each sample was spiked with 1.2 nmol of 15:0 fatty acid as internal standard immediately. For serum, 2.5 nmol of 15:0 was added to 50 μl of the sample followed by 0.5 ml of methanol. Then, lipids were extracted according to Bligh and Dyer method [25]. Lipid extracts were dried and dissolved in 50 μl of a pentafluoro benzyl bromide solution (3.4% in acetonitrile) and 10 μl of N, N-diisopropyl ethanolamine. After 10 min of incubation at room temperature samples were evaporated under a stream of nitrogen and resuspended in 50 μl of hexane. A Trace-DSQ GC–MS (Thermo Scientific, Austin, TX) equipped with a TR-FAME 30 m column was used in splitless mode with 1 ml/min helium as carrier gas and 300 °C injector temperature. The initial oven temperature of 150 °C was held for 1 min and then increased to 200 °C at a rate of 25 °C/min followed by 325 °C at a rate of 12.5 °C/min and held for 2 min. The mass spectrometer was run in negative ion chemical ionization (NICI) mode. Fatty acids were detected in full scan as carboxylates after loss of the pentafluoro benzyl moiety. Methane was used as CI gas. Source temperature was set to 250 °C and the transfer line temperature to 330 °C.

### 2.9 GC–EI/MS of total fatty acids (free and esterified)

Lipids were extracted according to Bligh & Dyer method as described above. For liver, each sample was spiked with 375 nmol of 15:0 fatty acid as internal standard. For serum, 22.5 nmol of 15:0 was added to 50 μl of the sample. Then lipids were dried and suspended in 1 ml methanol NaOH. After 10 min incubation at 80 °C, samples were cooled for 5 min on ice. Then, 1 ml BF_3_ was added and incubated for 10 min at 80 °C. Fatty acid methyl esters were extracted with 1 ml saturated NaCl and 2 ml hexane. The hexane phase was dried and methyl esters dissolved in 1.5 ml hexane. Trace-DSQ GC–MS equipped with a TR-FAME 30 m column was used for analysis. Helium was used as carrier gas at a flow of 1.3 ml/min, in split mode, at 250 °C injector temperature. Initial oven temperature of 150 °C was held for 0.5 min and then temperature was increased to 180 °C at a rate of 10 °C/min. This was followed by a further increase to 190 °C at a rate of 0.5 °C/min and then increased to 250 °C at a rate of 40 °C/min and kept for 3 min. The mass spectrometer was run in electron impact mode and fatty acids were detected in full scan of m/z 80–400. Source temperature was set to 250 °C and the transfer line temperature to 200 °C.

### 2.10 Hepatic TG determination

50–70 mg of liver samples was homogenized in methanol in MagNA Lyser (Roche). Lipids were extracted with chloroform/methanol/glacial acetic acid (66/33/1 v/v/v) and phase separation was achieved by addition of water. The lower organic phase was evaporated and reconstituted by brief sonication in 0.1% Triton X-100. For protein measurement, the tissue homogenates were solubilized in 0,3N NAOH/0.1% (w/v) sodium dodecylsulfate at 65 °C for several hours and the protein content was determined by using Bio-Rad Protein Assay (Lowry). TG content was measured with a commercial kit from Diagnostic Systems International (Holzheim, Germany).

### 2.11 RNA isolation and qRT-PCR analysis was performed as previously described [3]

mRNA levels were normalized to 36b4 as housekeeping gene. Sequences of primers used for qRT-PCR are available upon request. mRNA levels of inflammatory markers in WAT was analyzed from gonadal, perirenal and subcutaneous depots and only the gonadal WAT is shown (representative). Analysis of mRNA expression of other genes (except inflammatory markers) was performed from the stated depot.

### 2.12 Histology scoring

The liver tissues were fixed in 10% neutral buffered formalin and embedded in paraffin. 2 μm thick liver sections were stained with H&E or sirius red. Histological examination was performed in a blinded fashion by an experienced gastrointestinal board certified pathologist (Prof. Dr. Carolin Lackner) using the widely accepted Nonalcoholic Steatohepatitis Clinical Research Network (CRN) histological scoring system for NAFLD as described [26]. Briefly, steatosis and inflammation scores ranged from 0 to 3 with 0 being within normal limits and 3 being most severe. Individual scores were assigned for each parameter. The NAFLD activity score (NAS) was calculated from the sum of the individual scores for steatosis, inflammation and ballooning. The latter change was not observed and therefore scored as zero.

### 2.13 Statistics

Data are presented as mean ± SD of the reported animals in each group (4–6 animals). Statistical analysis was performed by using SPSS V.14.0. Statistical significance was determined by Student's unpaired two-tailed *t* test. Group difference was considered significant for p < 0.05 (*^, #, §^), p < 0.01(**^, ##, §§^), and p < 0.001 (***^, ###, §§§^).

## 3 Results

### 3.1 Effect of MCD diet on adipose depots and serum non-esterified fatty acid (NEFA) levels

**Fig. 1:**
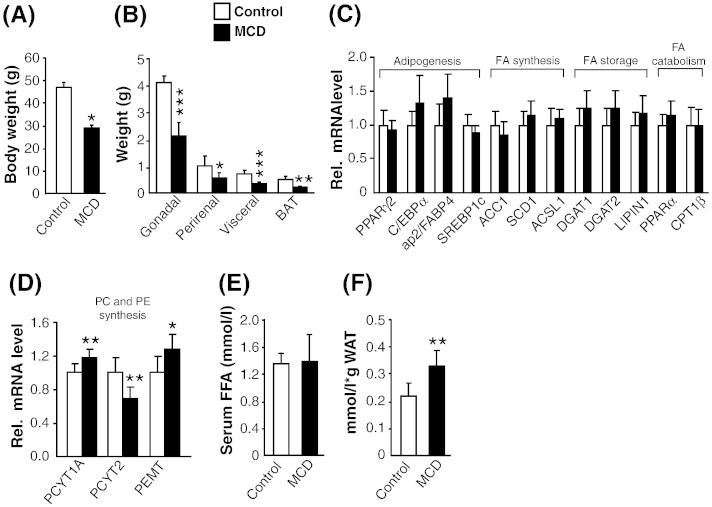
Effect of MCD diet on adipose tissue mass, its molecular markers and lipolysis. ob/ob Mice were fed with control or methionine–choline-deficient (MCD) diet for 5 weeks. Mice were harvested in non-fasted state. (A) Final body weight. (B) Fat depot analysis of gonadal, perirenal, visceral WAT and BAT. (C) mRNA expression of markers of adipogenesis, fatty acid (FA) synthesis, storage and catabolism in gonadal WAT. (D) mRNA expression of genes involved in phospholipid biosynthesis in gonadal WAT. (E) Serum NEFA concentration. (F) Serum NEFA concentration normalized to the total WAT weight. n = 6 per group. Bars represent mean + SD. ***p < 0.001 **p < 0.01, *p < 0.05, versus ob/ob control group.

To delineate the role of adipose tissue in steatohepatitis in a mouse model which combines key features of obesity and insulin resistance, we challenged ob/ob mice with MCD diet for 5 weeks to promote progression of steatosis to NASH. MCD-fed ob/ob mice lost 38% of their body weight at the end of 5 weeks (Fig. 1A) due to substantial reduction of all adipose depots: gonadal (− 48%), perirenal (− 44%), visceral (− 53%) and BAT (− 60%) (Fig. 1B) in line with the previous findings in MCD-fed db/db mice [27]. The expression of transcription factors known to promote adipogenesis, including, PPARγ2, C/EBPα, ap2/FABP4 and SREBP1c was similar in both control and MCD-fed mice (Fig. 1C) suggesting that defective adipogenesis was not responsible for WAT loss. Additionally, expression of genes involved in (i) FA synthesis, including ACC1, SCD1, ACSL1; (ii) FA storage, including DGAT1, DGAT2, LIPIN1 and (iii) FA catabolism, including PPARα and CPT1β were similar in both the groups which suggest that these processes of FA metabolism were not responsible for WAT loss in MCD-fed mice (Fig. 1C). Since choline deficiency may affect the phosholipid synthesis and thereby membrane structure and cellular function of the adipocyte, we analyzed genes involved in phosphotidylcholine (PC) and phosphotidylethanolamine (PE) synthesis. PCYT1A, involved in synthesis of PC from diacylglycerol was marginally increased (18%) in MCD-fed ob/ob mice (Fig. 1D, left). However, the ethanolamine-phosphate PCYT2 gene, involved in synthesis of PE from diacylglycerol was decreased by 30% (Fig. 1D, middle). Additionally, mRNA expression of PEMT, that catalyzes conversion of PE to PC, was marginally increased (27%) (Fig. 1D, right). Taken together, these data suggest a moderate shift towards an increase in de novo PC synthesis from diacylglycerol and PE. Despite substantial WAT loss, serum NEFA concentrations were not elevated (Fig. 1E). This apparent discrepancy may be attributed to the reduced amount of WAT in the MCD-fed group since NEFA levels were 1.5-fold elevated when normalized to the WAT weight (Fig. 1F).

### 3.2 Increased peripheral lipolysis in MCD-fed ob/ob mice is due to increased ATGL and HSL activity in adipose depots

**Fig. 2:**
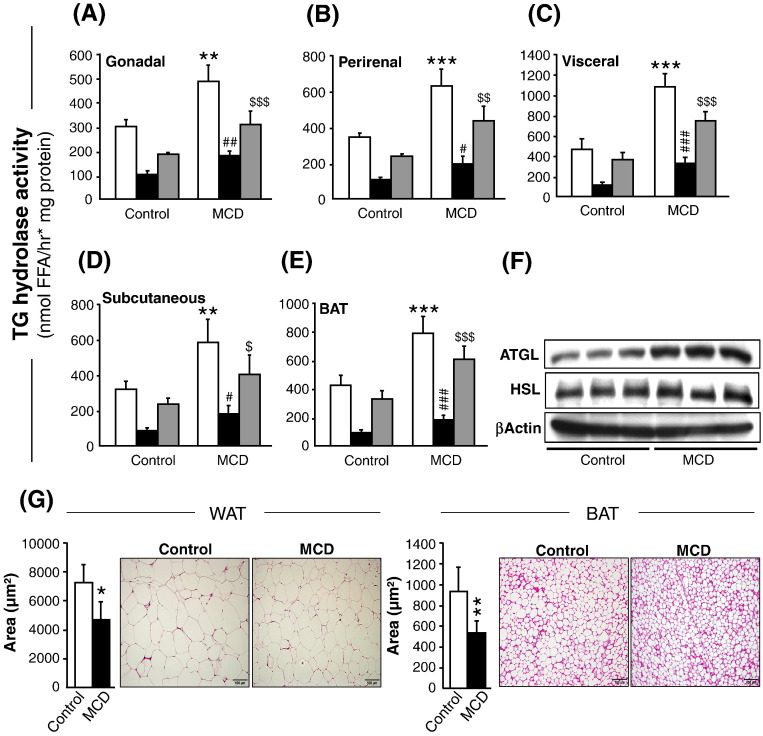
Adipose tissue TG hydrolase activity and its morphology in control- and MCD-fed ob/ob mice. (A–E) TG hydrolase activity in cytosolic extracts of gonadal (A), perirenal (B), visceral (C), subcutaneous (D) WAT and BAT (E). Total activity (white bars), ATGL activity (TG hydrolase activity after inhibition with 10 mM HSL inhibitor, black bars), HSL activity (determined by subtraction of HSL inhibited lipase activity from total lipase activity, gray bars). (F) Representative Western blots for protein levels of ATGL and HSL in subcutaneous WAT. (G) Adipocyte cell size analysis from Gonadal WAT (left) and BAT (right). Representative H&E stained sections show reduced adipocyte size from WAT and BAT of MCD-fed ob/ob mice. Magnification, 20x; Scale bar, 100 μm. n = 5–6 per group. Bars represent mean + SD. ^#,§^p < 0.05, **^,##,§§^p < 0.01, ***^,###,§§§^p < 0.001, versus corresponding ob/ob control group.

Loss of adipose depots and increased ratio of plasma free FAs per gram fat mass indicated an elevated lipolytic rate under non-fasting state. Indeed, as shown in (Fig. 2A–E, white bars), total TG hydrolase activity increased in all adipose depots with the highest increase observed in visceral (+ 130%), followed by perirenal (+ 83%), subcutaneous (+ 82%), gonadal WAT (+ 63%) and BAT (+ 86%). The maximal increase of ATGL activity (HSL-inhibited TG hydrolase activity) was seen in visceral (+ 204%), followed by subcutaneous WAT (+ 112%), BAT (+ 93%), perirenal (+ 82%) and gonadal WAT (+ 78%) (Fig. 2A–E, black bars). Compared to ATGL, HSL activity (determined by subtraction of HSL inhibited lipase activity from total lipase activity) was induced to a lesser extent by 108% in visceral, 71% in subcutaneous, 84% in BAT, 85% in perirenal and 65% in gonadal WAT (Fig. 2A–E, gray bars). In line, Western blot analysis also showed increased ATGL and to a lesser extent HSL protein expression in MCD-fed ob/ob mice (Fig. 2F). Collectively, these data show that MCD induces ATGL and HSL activities, resulting in loss of WAT and BAT depots in MCD-fed ob/ob mice. The physiological effect of enhanced lipolysis on morphology of adipose tissue was investigated by determining the adipocyte size from gonadal WAT and BAT. The fat cell size was very heterogeneous in all the areas, however the cell size decreased by − 35% and − 43% in WAT and BAT respectively in response to MCD feeding (Fig. 2G). Additionally, unlike the multilocular morphology of BAT, in ob/ob mice the BAT was unilocular in both the groups (Fig. 2G, right), as also shown previously [28].

### 3.3 Increased ATGL and HSL activity in adipose depots may be due to enhanced FGF21 levels

**Fig. 3:**
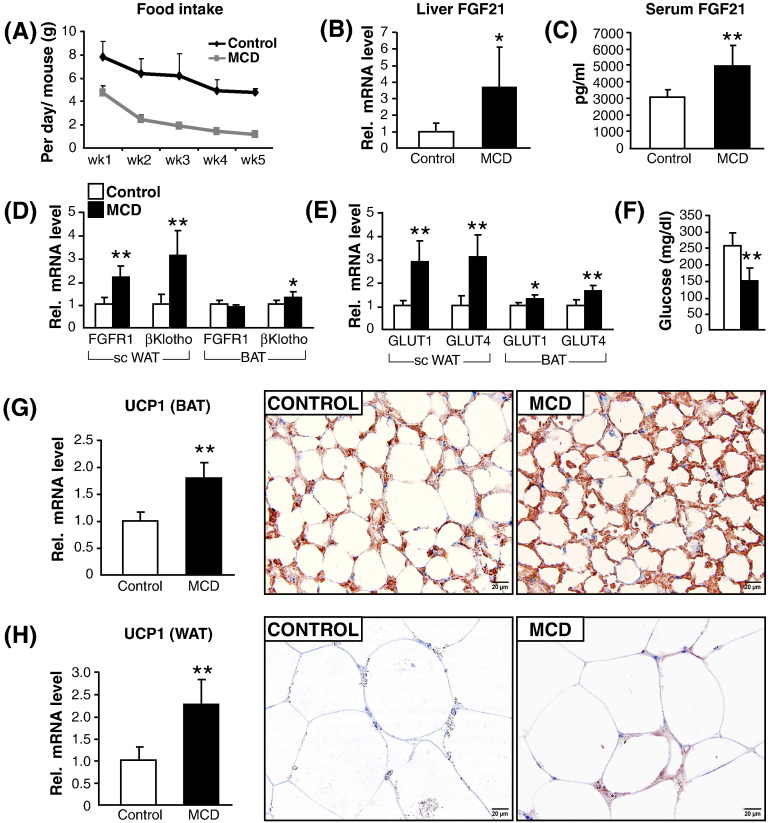
Enhanced FGF21 signaling and its effects on adipose depots. (A) Food intake during 5 week MCD feeding. (B) mRNA expression of FGF21 in liver. (C) Serum FGF21 concentration. (D) mRNA expression of FGF21 receptors in subcutaneous (sc) WAT and BAT. (E) mRNA expression of glucose uptake receptors in sc WAT and BAT. (F) Serum glucose concentration. (G) mRNA expression of UCP-1 (marker of increased FGF21 signaling and SNS outflow to adipose depots) in BAT (left); Representative immunohistochemistry (IHC) of UCP-1 showing, increased UCP-1 positive cells in BAT of MCD-fed ob/ob mice (right). (H) mRNA expression of UCP-1 in gonadal WAT (left); Representative IHC of UCP-1 showing detectable UCP-1 positive cells in gonadal WAT of MCD-fed ob/ob mice (right). Magnification, 60 ×; Scale bar, 20 μm. n = 5–6 per group. Bars represent mean + SD. **p < 0.01, *p < 0.05, versus ob/ob control group.

FGF21 is an endocrine hormone that plays a central role in energy homeostasis, substrate metabolism and fasting in rodents. Its administration reduces adiposity in mice [29–31]. Importantly, it induces lipolysis in adipose tissue via elevating ATGL and HSL mRNA and protein expression [31,32]. We therefore, hypothesized that increased lipase activity in MCD-fed ob/ob mice may be due to increased FGF21 signaling. Subsequently, we analyzed the systemic effect of FGF21 on WAT and BAT depots. With disease progression, MCD-fed ob/ob mice were no longer hyperphagic as also previously shown in db/db mice [27] (Fig. 3A). This may mimic a fasting-like response in these mice. In line, we observed an increase in FGF21 mRNA expression in liver (Fig. 3B). Importantly, serum FGf21 levels were increased by 60% in MCD-fed ob/ob mice (Fig. 3C). In line, with the well-established effect of FGF21 on subcutaneous (sc) WAT and BAT [29], the mRNA expression of FGF21 receptors- FGFR1 and βKlotho were increased by + 117% and + 208% respectively in sc. WAT (Fig. 3D). In BAT, the expression of FGFR1 was unchanged and βKlotho expression was increased by + 30% (Fig. 3D). Consequently, the expression of glucose transporter, GLUT1 and GLUT 4 was enhanced in sc. WAT (+ 185%, + 207%, respectively) and BAT (+ 30%, + 59%, respectively) and serum glucose levels were reduced by 42% (Figs. 3E and F). Subsequently, uncoupling protein-1 (UCP-1) expression which is also induced in line with a FGF21 effect [29] was increased in BAT (Fig. 3G) and WAT (Fig. 3H). However, its expression in WAT was very low compared to the BAT (Fig. 3G and H, right).

It has been previously shown, by separately feeding mice with either methionine- or choline-deficient diet that WAT loss by MCD feeding is due to the deficiency of methionine (and not choline) [8]. Additionally, dietary methionine restriction has been shown to reduce adiposity and increase energy expenditure both in humans and rodents [33–36]. The effect is via the Cyclic AMP (cAMP) signaling which represents the principal prolipolytic pathway in WAT and BAT, which is chiefly regulated by the SNS [23,37]. Dietary methionine restriction produces a chronic increase in SNS outflow to the adipose tissue producing a coordinated set of changes in WAT and BAT which limits fat deposition and increases energy expenditure [34]. Increased UCP-1 levels in BAT (Fig. 3G) and WAT (Fig. 3H) are also indicative of increase in SNS stimulation of adipose tissue [23,34,35,37]. Collectively, these findings suggest that MCD diet may induce WAT and BAT lipolysis (ATGL and HSL activity) through enhanced FGF21 signaling in addition to increased SNS outflow to the adipose depots.

### 3.4 Analysis of free/non-esterified fatty acid (NEFA) composition in serum and liver

**Fig. 4:**
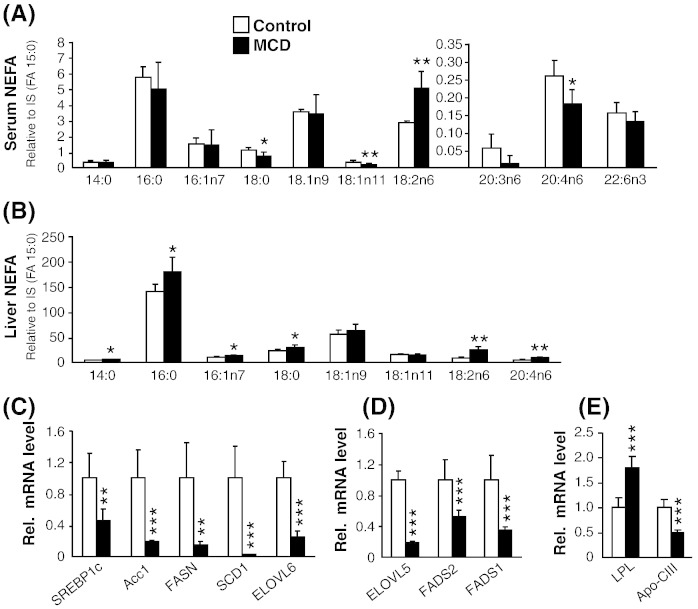
MCD feeding causes selective increase of non-esterified fatty acid (NEFA) species in serum and liver due to lipolytic flux from adipose tissue. (A and B) GC–NICI/MS profile of NEFA in serum (A) and liver (B). (C) Hepatic mRNA expression of genes involved in de-novo lipogenesis and monounsaturated fatty acid synthesis. (D) mRNA expression of genes involved in elongation and desaturation of polyunsaturated fatty acids. (D) mRNA expression of LPL and Apo-CIII. n = 6 per group. Bars represent mean + SD. ***p < 0.001 **p < 0.01, *p < 0.05, versus ob/ob control group.

Next, we determined NEFA composition in serum and liver of MCD- and chow-fed ob/ob mice by gas chromatographic/negative ion chemical ionization mass spectrometry (GC–NICI/MS). 18:2n6 (linoleic acid), was the major free FA species found to be significantly elevated (+ 89%) in serum of MCD-fed mice. While there was a modest decrease in fatty acids – 18:0, 18:1n11 and 20:4n6 (Fig. 4A). GC–NICI/MS of NEFAs in liver showed a marginal but significant increase in the saturated fatty acids – 14:0 (+ 17%), 16:0 (+ 28%), and 18:0 (+ 30%), (Fig. 3B). With respect to the monounsaturated fatty acid (MUFA), only 16:1n7 was significantly increased (+ 27%) in MCD-fed mice while there was no significant change in the levels of 18:1n9 and 18:1n11. While the polyunsaturated fatty (PUFA) acid – 18:2n6 showed the maximal increase (+ 195%) followed by 20:4n6 (+ 90%) in MCD-fed ob/ob mice (Fig. 4B).

We next analyzed whether the increased levels of these fatty acids in liver were due to increased lipolytic flux from the adipose tissue or due to increase in DNL or elongation and desaturation of fatty acids in the liver. Our analysis of mRNA expression revealed markedly reduced expression of genes involved in DNL and MUFA synthesis, including sterol regulatory element-binding protein 1c (SREBP1c, − 55%), acetyl CoA carboxylase 1 (Acc1, − 81%), fatty acid synthase (FASN, − 85%), stearoyl-CoA desaturase-1 (SCD1, − 98%) and fatty acid elongase 6 (ELOVL6, − 76%) in MCD-fed ob/ob mice (Fig. 4C), suggesting that the increased levels of FAs – 14:0, 16:0, 18:0 and 16;1n7 – were the contribution from WAT. Increased level of 18:2n6 in liver was the direct contribution from AT since it is an essential FA. 20:4n6 is also the elongation and desaturation product of 18:2n6 by fatty acid elongase – ELOVL5 – and fatty acid desaturases – FADS2 and FADS1. However, the expression of these genes was all down-regulated in MCD-fed ob/ob mice by 48–82% (Fig. 4D), suggesting that increased level of 20:4n6 was the contribution from WAT. Additionally, mRNA level of lipoprotein lipase (LPL) was markedly elevated (+ 79%) while its inhibitor, Apo-CIII was reduced (− 50%) in MCD-fed ob/ob livers, which may implicate an additional role for increased LPL-mediated FA uptake by the liver (Fig. 4E).

### 3.5 Effect of MCD diet on total fatty acid (TFA) composition in serum and liver and TG hydrolase activity in liver

**Fig. 5:**
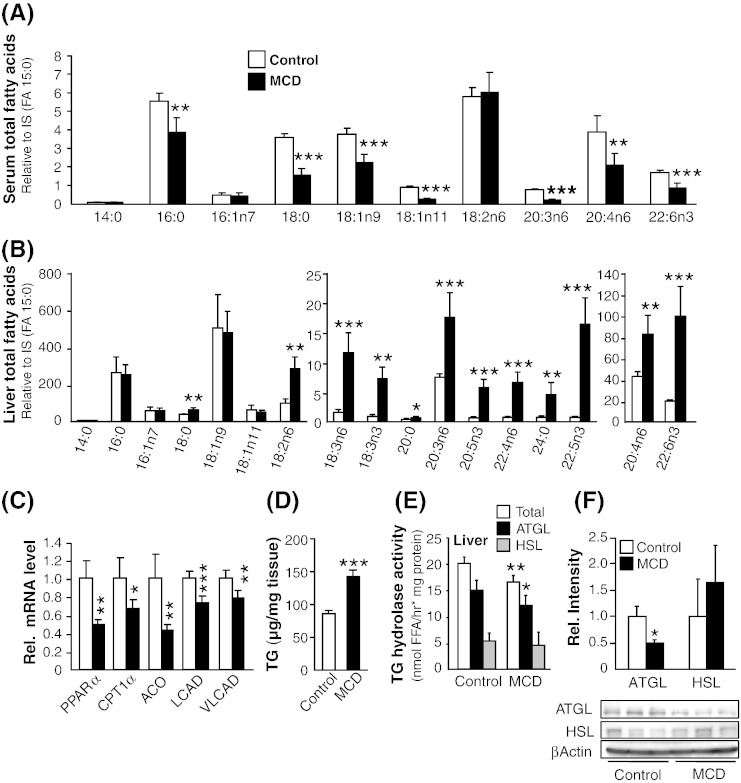
MCD feeding changes the homeostasis of total fatty acid (TFA) profile in serum and liver. (A and B) GC/MS profile of TFA in serum (A) and liver (B). (C) mRNA expression of genes involved in FA oxidation. (D) Hepatic triglyceride quantification. (E) TG hydrolase activity in liver. Total activity (white bar), ATGL activity (black bar), HSL activity (gray bar). n = 6 per group. (F) Western blot analysis of ATGL and HSL protein levels in liver of control and MCD-fed ob/ob mice. n = 3. Bars represent mean + SD. ***p < 0.001 **p < 0.01, *p < 0.05, versus ob/ob control group.

Since fatty acids are predominantly present/stored in the esterified form, we therefore analyzed TFA (all FAs from triglyceride, cholesterol ester or phospholipid pool) composition in serum and liver by GC–MS. Serum analysis showed overall reduced TFA composition (Fig. 5A) whereas, analysis of TFA composition in the liver showed an overall increase of all PUFAs, while 18:0 was the only saturated FA elevated in ob/ob MCD-fed livers (Fig. 5B). In line, our analysis of mRNA expression in liver revealed reduced expression of genes involved in fatty acid oxidation, including peroxisome proliferator-activated receptor α (PPARα, − 51%), carnitine palmitoyltransferase 1α (CPT1α, − 33%), acyl-CoA oxidase (ACO, − 57%), long-chain acyl Co-A dehydrogenase (LCAD, − 27%) and very long-chain acyl Co-A dehydrogenase (VLCAD, − 23%) (Fig. 5C). Subsequently, liver TG content was increased by 2-fold in MCD-fed ob/ob mice (Fig. 5D). In contrast to the increased total lipase activity in WAT of MCD-fed ob/ob mice, in liver, total lipase activity decreased by − 18% (Fig. 5E, white bar). ATGL activity was lowered by − 19% in MCD-fed ob/ob mice, while no change in HSL activity was observed (Fig. 5E, black and gray bars respectively). In line, western blot analysis showed significant decrease in protein expression of ATGL whereas HSL expression remained unchanged (Fig. 5F). Collectively, these findings suggest that in addition to reduced VLDL synthesis and secretion from the liver (data not shown) [38] hepatic fat accumulation in MCD-fed ob/ob mice may result from the combined effects of flux of free FAs from adipose tissue depots to the liver, reduced TG hydrolase activity and FA oxidation in the liver.

### 3.6 Effect of MCD diet on liver injury and NAFLD activity score (NAS)

**Fig. 6:**
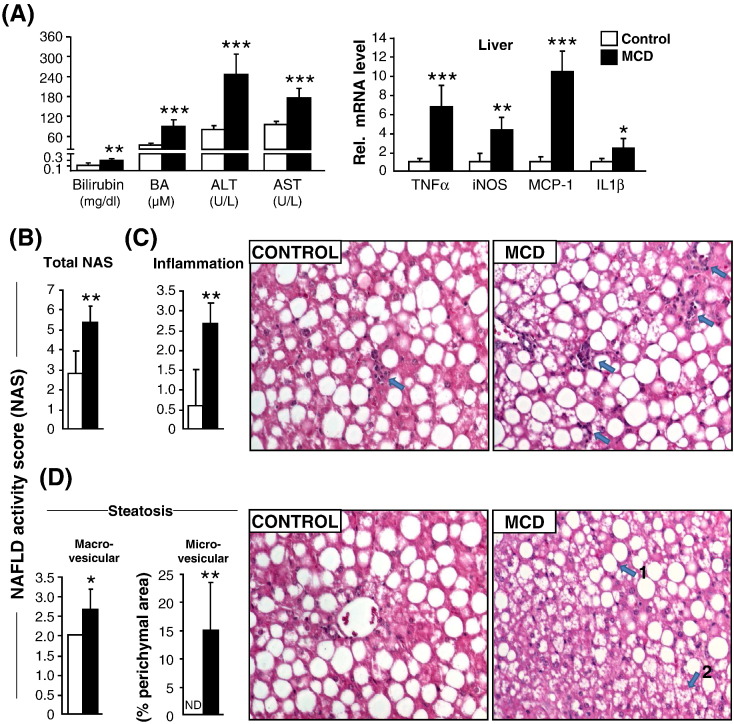
MCD feeding for 5 weeks enhances liver injury and NAFLD activity score (NAS). (A) Serum parameters of liver injury (left). mRNA expression of pro-inflammatory markers in liver (right). (B) NAS score (calculated from individual scores for steatosis + lobular inflammation and ballooning). (C) Individual scores for lobular inflammation were significantly higher in MCD-fed mice compared to controls (left); Representative H&E picture showing mild lobular inflammation (arrow) in the control group (left), whereas there are several foci of lobular inflammation (arrows) in the MCD group (right). (D) Individual scores for macrovesicular steatosis were also significantly higher in the MCD fed group as compared to the controls. (left). Microvesicular steatosis was only detected in MCD-fed mice but not in controls (middle). Representative H&E picture showing liver parenchyma with macrovesicular steatosis in the control group, whereas macrovesicular (arrow 1) and microvesicular (arrow 2) steatosis was present in the MCD group (right). Magnification, 40 ×;. n = 6 per group. Bars represent mean + SD. ***p < 0.001 **p < 0.01, *p < 0.05, versus ob/ob control group.

Following massive fatty acid flux from adipose tissue, MCD feeding resulted in pronounced liver injury as reflected by increased serum levels of bilirubin, bile acids (BAs), alanine aminotransferase (ALT) and aspartate aminotransferase (AST) (Fig. 6A, left) together with hepatic inflammation reflected by increased mRNA expression of tumor-necrosis factor-alpha (TNFα), inducible nitric oxide synthase (iNOS), monocyte chemotactic protein-1 (MCP-1), and interleukin 1beta (IL1β) (Fig. 6A, right). In addition, NAS scoring, performed by pathophysiological examination revealed significantly higher scores in MCD-fed group as compared to the controls (Fig. 6B). In line, histological scores for inflammation (Fig. 6C) and steatosis (Fig. 6D) were also high. MCD-fed group showed microvesicular in addition to macrovesicular steatosis (Fig. 6D, left). Ballooning was not clearly detected in this study. Fibrosis was also not detected since leptin deficient ob/ob mice are resistant to fibrosis [39].

### 3.7 MCD feeding attenuates inflammatory markers in BAT but not WAT

**Fig. 7:**
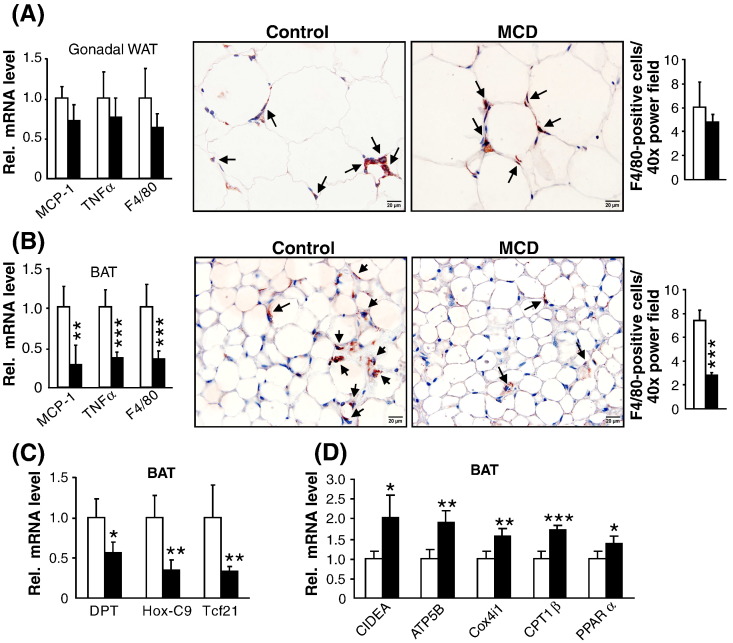
MCD feeding does not affect inflammatory markers in WAT but reduces inflammation in BAT. (A) mRNA expression of pro-inflammatory markers in gonadal WAT (left); Representative F4/80 IHC (middle) and its quantification (right) shows similar number of positive cells in gonadal WAT of control and MCD-fed mice. (C) mRNA expression of pro-inflammatory markers in BAT (left); Representative F4/80 IHC (middle) and its quantification (right) shows decrease in number of positive cells in MCD-fed ob/ob mice. Magnification, 60 ×; Scale bar, 20 μm. (D) mRNA expression of WAT enriched genes in BAT. (E) mRNA expression of markers of energy expenditure, thermogenesis and FA oxidation. n = 6 per group. Bars represent mean + SD. ***p < 0.001 **p < 0.01, *p < 0.05, versus ob/ob control group.

mRNA expression analysis from WAT did not show any difference in inflammatory markers between the two groups (Fig. 7A, left). To determine whether these changes of inflammatory markers were due to alteration in gene expression or to a change in macrophage number, we performed IHC using an antibody that recognizes the macrophage antigen F4/80. Consistent with the gene expression studies, macrophage number in gonadal adipose tissue remained unchanged in both groups (Fig. 7A, middle and right).

In contrast to WAT, BAT showed even reduced expression of inflammatory markers in MCD-fed ob/ob mice (Fig. 7B, left). In line, the macrophage number (F4/80) decreased by − 63% in MCD-fed ob/ob mice (Fig. 7B, middle and right). Our analysis of mRNA expression in BAT revealed markedly reduced levels of WAT enriched genes including dermatopontin (DPT, − 43%), homeobox protein C9 (Hoxc9, − 65%) and transcription factor 21 (Tcf21, − 67%) in MCD-fed mice (Fig. 7C). Furthermore, expression of genes involved in energy expenditure, thermogenesis, mitochondrial and peroxisomal FA oxidation, such as UCP-1 (Fig. 3G), CIDEA, ATP5B, COXIV, CPT1β, PPARα and PHYH were all increased by 35–100% in BAT of MCD-fed ob/ob mice (Fig. 7D). Together, these data in addition to the increased lipase activity in BAT (Fig. 2E) suggest that MCD diet reduces adiposity in BAT and enhances its activity and oxidative capacity which may be responsible for reduced inflammation in BAT.

## 4 Discussion

In the present study, we have examined the specific contribution of adipose tissue in causing hepatic steatosis and inflammation as key steps in the progression of NASH. Our data provides compelling evidence to show that increased ATGL and HSL activity in adipose tissue causes changes in FA homeostasis in serum and liver of MCD-fed mice and consequently leads to hepatic steatosis and lipotoxicity. Enhanced FGF21 signaling and its influence on adipose depots may partly be responsible for increased lipase activity in adipose depots. We also show that MCD diet augments BAT activity and reduces its inflammatory markers in ob/ob mice.

MCD diet is a widely used dietary model of NASH in mice which results in liver injury resembling human NAFLD/NASH and is increasingly used to obtain insights into the pathogenesis of NASH although several shortcomings such as weight loss, lack of insulin resistance and obesity in this model have to be acknowledged [3–5,27]. Therefore, we combined MCD diet with ob/ob mice as an established model of NAFLD. The contribution of WAT in the pathogenesis of NASH in this model has not been studied, despite its significant role in NAFLD patients [13]. Earlier studies have shown weight and WAT loss on MCD feeding [27,40], but the underlying mechanisms have remained unclear. In humans, increased peripheral FA flux has been shown to contribute ~ 59% to the hepatic TG content as opposed to ~ 26% by DNL and ~ 15% from the diet in NAFLD patients [13]. Our study now demonstrates that when applied to ob/ob mice on MCD diet as a murine model of NASH, increased ATGL and HSL activities resulted in massive loss of all WAT depots, thus increasing the flux of FAs from adipose tissue to liver. As such, the MCD model mimics the hepatic stress resulting from FA flux to the liver, in addition to its potential direct impact on liver causing oxidative stress as a result of micronutrient deficiency [8,9]. Moreover, the WAT loss observed under MCD feeding may also reflect part of the pathomechanisms resulting in steatosis and NASH under severe calorie restriction and massive weight loss after bariatric surgery [18,19,41,42] in addition to WAT mobilization seen in insulin-resistance states.

We have earlier shown that short-term MCD feeding or cancer-induced cachexia increases lipase activity and causes WAT loss in WT and HSL-KO mice and that ATGL-KO mice are partially protected from WAT loss [3,43]. Based on these findings, together with our current observations, it is quite conceivable that ATGL may be the rate limiting metabolic lipase responsible for increased FA flux from adipose tissue in human NAFLD/metabolic syndrome patients also, as it is for patients suffering from cancer-induced cachexia [43]. Notably, various WAT depots behaved differently in terms of lipolytic activity mediated fat loss, with visceral WAT showing the highest rate of mobilization and subcutaneous the least. These observations in mice are in line with lipolysis from WAT as a major source of FA in humans where visceral WAT has a higher rate of mobilization compared to subcutaneous WAT [44]. Furthermore, venous blood from visceral fat is directly drained to the liver through the portal vein. Therefore visceral fat is metabolically more active and is the major contributing source in the overflow of FAs to the liver [13]. Notably, expansion of visceral but not subcutaneous fat depots has been linked to insulin resistance and NAFLD [45].

It has been previously shown that reduction in body weight after MCD feeding is a characteristic of methionine deficiency [8] and was not associated with the biochemical effect of fasting [46]. Moreover, WT mice on MCD diet consumed similar amount of food as their controls [3,40,46,47]. Thus it would be incorrect to conclude that adipose tissue loss is related entirely to reduced food intake. It has been shown that FGF21 acts via activation of BAT and browning of WAT, leading to increased glucose uptake and energy metabolism [29]. While our results are in line in these effects of FGF21 in adipose tissue, a clear indication of browning of WAT was not observed at the level of gene expression and WAT morphology (data not shown). FGF21 gene expression in WAT and BAT per se was not increased (data not shown), but the increase in expression of its receptors in these depots suggests that the FGF21-mediated effect in adipose depots may be due to the enhanced systemic levels of FGF21. Based on these findings, together with the previous reports of FGF21 action on adipose tissue [29–32], it is quite conceivable that part of the mechanism for increased lipase activity in adipose tissue is mediated via FGF21. Alternatively, increased SNS activation-βadrenergic stimulation-mediated increase in lipase activity due to dietary methionine restriction may also be involved [23,34,35,37]. Similarly, deprivation of another essential amino acid – leucine, decreased fat mass by stimulation of lipolysis in WAT and upregulation of UCP-1 in BAT [48]. Our present data on serum FGF21 levels is also in line with the earlier study in humans which showed that hepatic FGF21 mRNA expression was significantly elevated in NAFLD patients and it correlated with a substantial increase in serum FGF21 in these patients [49].

Our data demonstrates the importance of separately analyzing different FA pools, i.e total FA (free and esterified) and free FA/NEFA. Analysis of NEFA in serum revealed that 18:2n6 was preferentially released from adipose tissue into the serum. This indicates that esterified-18:2n6 could be one of the preferred substrates of ATGL. Indeed it has been shown earlier that ATGL preferentially hydrolyses long-chain FA esters in vivo (upon 8 h fasting) [50]. Increased level of non-esterified 18:2n6 (+ 195%) in liver (Fig. 4B) was the direct contribution from AT (Fig. 4A) which needs to be esterified into TG's and again rehydrolysed by ATGL before they can become active signaling ligands for PPARα [51]. Subsequently, esterified 18:2n6 was also increased in the liver by 193% (Fig. 5B) which implies that 18:2n6 was unavailable for PPARα activation because it was not hydrolysed by ATGL due to reduced ATGL activity (Fig. 5E). Therefore, despite increased levels of important PPARα ligands – 18:2n6 and 20:4n6 – in the liver, MCD-fed ob/ob mice showed attenuated PPARα signaling and enhanced inflammation. The massive increase of overall TFA composition in the liver of MCD-fed ob/ob mice reflects the combined effect of increased fatty acid flux from adipose tissue, decreased oxidation of fatty acids and reduced TG hydrolase activity in the liver. Excess FA accumulation may further sensitize the liver for injury and inflammation. Further, the lack of FA hydrolysis and oxidation, limits the ligand availability for PPARα signaling as well as other cellular signaling processes dependent on lipolytic products and their intermediates [22]. PPARα insufficiency further leads to aggravation of NASH [52] which is accentuated in ATGL-KO mice due to the lack of ATGL-mediated generation of FAs as ligands for PPARα [3]. In contrast to the NEFA profile in serum and liver, the TFA profile in serum showed an overall decreased composition of FA species which may be attributed to the reduced lipoprotein synthesis and excretion from the liver [7].

A previous human study showed that impaired BAT activity or the absence of it in adult life is associated with an increased risk of fatty liver [53], similar to associations found in obesity and diabetes mellitus patients [54]. While reduced BAT mass in MCD-fed ob/ob mice is in line with the human data, increased BAT activity contrasts this preliminary human study. Further studies are required to elucidate these observations in both humans and rodents.

It would be ideal to conduct pair-feeding experiments to match weight loss in control and MCD-fed ob/ob mice, although such is the severity of weight loss in MCD-fed mice that this would be technically very challenging. Our study was focused on a relatively late stage of MCD feeding and therefore it is plausible that in the early stages there may be more contribution of DNL to hepatic steatosis. Whether blocking peripheral lipolysis could limit hepatic steatosis and further progression to NASH remains to be determined. However, since AT may also provide ligands for PPARα activation in the liver, blocking peripheral lipolysis may also exaggerate the inflammatory response in progression to NASH. We have previously shown that despite partial protection from WAT loss (40–50%), genetic ablation of ATGL leads to pronounced hepatosteatosis upon MCD feeding. This finding strongly suggests the role of other yet unknown lipase(s) which need to be further investigated [3]. Importantly, our findings from the present and previous study imply that the role of ATGL in the liver is imperative for TG hydrolysis because the bottleneck is the reduced ATGL activity in the liver, followed by reduced oxidation of fatty acids which causes hepatosteatosis. This effect is further accentuated in ATGL-KO mice [3].

In conclusion, our data support a key role for increased ATGL and HSL activity in adipose tissue, leading to increased FA delivery and TG production in the liver in conjunction with a compromised capacity of the liver to compensate with increased hepatic lipolysis, FA oxidation and TG export in this model of NASH. Our findings demonstrate an important conceptual advance in understanding the mechanisms by which MCD diet causes hepatic steatosis and inflammation by mimicking the shift of FA/TG partitioning from WAT to the liver to mimic this important aspect in the pathogenesis of NASH.

## Funding

This work was supported by grants F3008 (to MT) from the Austrian Science Foundation and the European Community's Seventh Framework Program (FP7/2007–2013) under grant agreement HEALTH-F2-2009-241762 for the project FLIP (to MT). P. Jha was also supported by the PhD program of the Medical University of Graz.
